# PKCδ serves as a potential biomarker and therapeutic target for microglia‐mediated neuroinflammation in Alzheimer's disease

**DOI:** 10.1002/alz.14047

**Published:** 2024-06-28

**Authors:** Ying Du, Tiantian Guo, Yunfeng Hao, Chuan Li, Linghui Tang, Xia Li, Xiaoxiao Zhang, Lin Li, Dan Yao, Xia Xu, Huaxing Si, Jinghan Zhang, Nana Zhao, Tong Yu, Yingjun Zhao, Wei Zhang, Huaxi Xu

**Affiliations:** ^1^ Department of Neurology Tangdu Hospital Fourth Military Medical University Xi'an Shaanxi China; ^2^ Center for Brain Sciences the First Affiliated Hospital of Xiamen University Institute of Neuroscience Fujian Provincial Key Laboratory of Neurodegenerative Disease and Aging Research School of Medicine Xiamen University Xiamen Fujian China; ^3^ Department of Anatomy Histology and Embryology and K. K. Leung Brain Research Centre Fourth Military Medical University Xi'an Shaanxi China; ^4^ Department of Respiratory and Critical Care Medicine Xijing Hospital Fourth Military Medical University Xi'an Shaanxi China; ^5^ College of Life Science Northwest University Xi'an Shaanxi China; ^6^ Department of Neurology the Second Affiliated Hospital of Shaanxi University of Chinese Medicine Xianyang Shaanxi China

**Keywords:** Alzheimer's disease, microglia, neuroinflammation, NF‐κB, PKCδ

## Abstract

**INTRODUCTION:**

To investigate the role of a novel type of protein kinase C delta (PKCδ) in the neuroinflammation of Alzheimer's disease (AD).

**METHODS:**

We analyzed PKCδ and inflammatory cytokines levels in cerebrospinal fluid (CSF) of AD and normal controls, as well as their correlations. The cellular expression pattern of PKCδ and the effects of PKCδ modulation on microglia‐mediated neuroinflammation were evaluated by quantitative real‐time polymerase chain reaction (qRT‐PCR), western blot, RNA sequencing (RNA‐seq), and immunofluorescence staining.

**RESULTS:**

PKCδ levels were increased dramatically in the CSF of AD patients and positively correlated with cytokines. PKCδ is expressed mainly in microglia in the brain. Amyloid beta (Aβ) stimulation increased PKCδ expression and secretion, which led to upregulation of the nuclear factor kappa B (NF‐κB) pathway and overproduction of proinflammatory cytokines. Downregulation or inhibition of PKCδ attenuated Aβ‐induced microglial responses and improved cognitive function in an AD mouse model.

**DISCUSSION:**

Our study identifies PKCδ as a potential biomarker and therapeutic target for microglia‐mediated neuroinflammation in AD.

**Highlights:**

- Protein kinase C delta (PKCδ) levels increase in cerebrospinal fluid (CSF) of patients with Alzheimer's disease (AD), and positively correlate with elevated inflammatory cytokines in human subjects.
- PKCδ is expressed mainly in microglia in vivo, whereas amyloid beta (Aβ) stimulation increases PKCδ expression and secretion, causing upregulation of the nuclear factor kappa B (NF‐κB) pathway and production of inflammatory cytokines.
- Downregulation or inhibition of PKCδ attenuates Aβ‐enhanced NF‐κB signaling and cytokine production in microglia and improves cognitive function in AD mice.
- PKCδ serves as a potential biomarker and therapeutic target for microglia‐mediated neuroinflammation in AD.

## 1 BACKGROUND

Alzheimer's disease (AD) is the most common type of dementia associated with progressive cognitive decline and memory loss in the elderly worldwide. Although the exact etiology of AD is not fully understood, accumulating evidence has demonstrated that overproduction of amyloid beta (Aβ) peptide and the accompanied neuroinflammation plays a key role in the pathogenesis of AD.^1^ Available treatments for AD so far only partially manage clinical symptoms, and there is a need for therapeutics halting or reversing Aβ‐induced neuroinflammation and cognitive impairment in the disease.^2^, ^3^, ^4^

Microglia are the primary innate immune cells in the central nervous system (CNS), which maintain normal brain function via immune surveillance. Microglia are chronically overactivated by accumulated Aβ during AD pathogenesis and can trigger prominent neurotoxicity in the CNS through secreting pro‐inflammatory factors such as interleukin 1 beta (IL‐1β), interleukin 6 (IL‐6), and tumor necrosis factor alpha (TNF‐α). These inflammatory cytokines can not only promote Aβ production, but also reciprocally facilitate microglial response to Aβ, thereby forming a vicious cycle between Aβ‐induced pathogenesis and microglia‐mediated neuroinflammation.^5^, ^6^, ^7^, ^8^ However, the key factors that drive microglial inflammation in AD have not been fully elucidated.

Protein kinase C delta (PKCδ) belongs to the novel PKC isozyme family and is widely expressed in cortical and hippocampal regions of the brain. As a serine/threonine kinase, PKCδ exerts its function mainly via the downstream pathways, such as nuclear factor kappa B (NF‐κB) pathway.^9^, ^10^, ^11^, ^12^ We recently have shown that the expression of PKCδ is increased in post‐mortem brain samples of AD patients and that neuronal PKCδ can regulate Aβ production mediated by β‐site amyloid precursor protein (APP)‐cleaving enzyme 1 (BACE1).^13^ Of interest, data from single‐cell sequencing (GSE129788) show enriched expression of PKCδ in the microglia.^14^ However, the precise role of PKCδ in microglial function and AD‐associated neuroinflammation remains poorly understood.

In the current study, we determined the association between PKCδ and inflammatory cytokines in the cerebrospinal fluid (CSF) of AD patients, as well as the correlation of CSF PKCδ with cognitive function in our cohort. In addition, we investigated how PKCδ regulates microglia‐mediated inflammation in cellular and AD mouse models.

## 2 METHODS

### 2.1 Standard protocol approvals

This study was performed according to the Declaration of Helsinki and approved by the ethical committee of Tangdu Hospital, Fourth Military Medical University. Written informed consent was obtained from all participants or their legal representatives. All animal studies were performed according to the protocols approved by the Institutional Animal Care and Use Committee (IACUC) of Tangdu Hospital, Fourth Military Medical University. Experiments were conducted and analyzed in a double‐blind manner.

### 2.2 Study population

The study recruited 32 Chinese patients with AD diagnosed in the Tangdu Hospital Department of Neurology from April 2020 to April 2021. All patients with AD met the following inclusion criteria: (1) dementia based on the criteria of the National Institute on Aging and Alzheimer's Association (NIA‐AA); (2) CSF was acquired by lumber puncture for AT(N) biomarker analysis and presenting as A+T+(N+) according to cutoff values currently used in each memory clinic,^15^, ^16^, ^17^, ^18^, ^19^ decreased Aβ1‐42 labeled “A+” for aggregated Aβ, increased phosphorylated tau 181 (p‐Tau181) labeled “T+” for phosphorylated tau, and increased total tau (t‐Tau) labeled “N+” for neurodegeneration.^20^ The exclusion criteria were: (1) multi‐modal magnetic resonance imaging (MRI) scan showing cerebral infarction, hemorrhage and microbleeds, subdural hematoma, leukoaraiosis, and normal pressure hydrocephalus (NPH); (2) concomitant neurological or mental disorders potentially affecting cognitive function, such as rapidly progressive dementia and Parkinson's syndrome; (3) meeting the clinical diagnostic criteria for frontotemporal lobar degeneration (FTLD), dementia with Lewy body (DLB), and vascular dementia (VaD); (4) AT(N) biomarkers in CSF presenting A‐T ± (N) (Aβ pathology negative, regardless of the presence or absence of tau pathology and neurodegeneration); and (5) other comorbidities, such as malignant tumors, poisoning, heredity, trauma, syphilis, AIDS, thyroid dysfunction, hyper‐homocysteinemia, and vitamin B12 deficiency. In addition, an age‐ and gender‐matched cohort of 35 participants with normal cognition and A‐T‐(N) (Aβ and tau pathology negative, and without neurodegeneration) in CSF was also included (normal cognition (NC) group) (Figure S1).

### 2.3 Clinical assessment

All the participants were subjected to medical examination and cognitive assessment by experienced neurologists. Demographic and medical data were also collected. As described previously, the Mini‐Mental Status Examination (MMSE) scale consists of 30 questions with the highest points at 30, and tests five cognitive domains, including time and place orientation (10 points), memory registration (3 points) and recall (3 points), attention and calculation (5 points), and language and praxis (9 points). Higher scores indicate better cognition. Specifically, both “MMSE ≤24 with high school or above” and “MMSE ≤20 with below high school” are considered as dementia.^21^ The Neuropsychiatric Inventory (NPI) scale consists of 12 items of neuropsychiatric disturbances common in dementia, including delusions, hallucinations, agitation, dysphoria, anxiety, apathy, irritability, euphoria, disinhibition, aberrant motor behavior, night‐time behavior disturbances, and appetite and eating abnormalities. The severity and frequency of each symptom are rated on the scripted questions for the patient's caregiver, and then followed by a calculation of total NPI score.^22^ The Activities of Daily Living (ADL) scale consists of 9 items of basic ADL and 11 items of instrumental ADL (scored 0 to 80; higher scores indicate worse impairment, carer rated).^23^

**RESEARCH IN CONTEXT**

1. **Systematic review**: The authors reviewed the literature using PubMed and Google. Neuroinflammation induced by amyloid beta (Aβ) has been linked to progressive neurodegeneration in Alzheimer's disease (AD), where the underlying mechanisms remain to be fully defined. A novel type of protein kinase C delta (PKCδ) has been recently indicated to regulate neuronal Aβ generation and deposition. However, the role of PKCδ in AD‐associated neuroinflammation remains largely unclear.
2. **Interpretation**: We first reported dramatically increased PKCδ levels in the CSF of patients with AD, and identified positive correlations of PKCδ and cytokines in human subjects. Data from cellular and animal studies demonstrate that PKCδ is expressed mainly in microglia and Aβ stimulation increases PKCδ expression and secretion. Moreover, downregulation or inhibition of PKCδ attenuates Aβ‐enhanced NF‐κB signaling and cytokine production in microglia and improves cognitive function in AD mice. These data indicate that PKCδ is a potential biomarker and therapeutic target for microglia‐mediated neuroinflammation in AD.
3. **Future directions**: Large cohort studies are needed to confirm clinical practicality of PKCδ as a biomarker for AD diagnosis. PKCδ functions in the microglia need to be further characterized in genetic mouse models with microglial deletion or upregulation of PKCδ. Whether and how PKCδ affects tau pathogenesis should also be examined.

### 2.4 CSF sampling and protein levels analysis

CSF was collected with polypropylene syringes using a Sprotte 25‐gauge spinal needle in an intervertebral lumbar space. Samples were immediately transported to the laboratory for centrifugation (1800 × *g*, 20°C, 10 min). The supernatant was mixed gently to avoid possible gradient effects, divided into aliquots in the polypropylene tubes, and stored at ‐80°C pending biochemical analysis. Samples were measured using commercially available enzyme‐linked immunosorbent assays (ELISAs) for CSF Aβ1‐42 (Fujirebio Europe, Gent, Belgium), p‐Tau181 (Fujirebio Europe N.V., Belgium) and t‐Tau (Fujirebio Europe, Gent, Belgium), as well as PKCδ (LifeSpan Biosciences, Inc), IL‐1β, IL‐6, and TNF‐α (R&D Systems, Inc).^24^, ^25^, ^26^

### 2.5 Preparation of Aβ oligomers

Aβ1‐42 peptides (Anaspec) were dissolved in hexafluoroisopropanol (HFIP) and subsequently dried using a SpeedVac system (Thermo Fisher Scientific). The lyophilized Aβ was dissolved in dimethyl sulfoxide (DMSO), sonicated for 10 min, diluted in phosphate‐buffered saline (PBS) to 100 µM, and then oligomerized by incubation at room temperature (RT) for 48 h in DMSO/PBS, as described previously.^3^

### 2.6 Primary microglial culture and treatment

Primary microglial culture was prepared according to a protocol described previously, with some modifications.^27^ Cortical and hippocampal tissues were isolated from mouse on postnatal days 1–2 and mechanically crushed to generate cell suspension. Cells were resuspended in DMEM (catalog #11965‐092, Gibco) containing 20% fetal bovine serum (FBS), 1% penicillin/streptomycin, and 25 ng/mL granulocyte‐macrophage colony‐stimulating factor (catalog #315‐03, Peprotech), and then seeded into 175 cm^2^ flasks coated with 0.1% poly‐L‐lysine (catalog #P6282, Sigma‐Aldrich) for proliferation of mixed glial cells. Primary microglia were collected by shaking (200 rpm, 30 min) 10–12 days after culture and every 3 days thereafter (up to four or five times).

For some experiments, primary microglia were treated with 100 ng/mL lipopolysaccharide (LPS) for 6 h or 1 µM Aβ42 oligomers for 12 h. To examine secreted proteins, the complete medium of primary microglial culture was replaced with serum‐free medium, and supernatants were collected after 6 h. The conditioned medium was incubated with 10% trichloroacetic acid and the precipitated proteins were collected via centrifugation at 20,000 × *g*, 15 min, and then subjected to western blot assays.

To overexpress PKCδ, primary microglia were transduced with the recombinant lentivirus expressing mouse PKCδ (LV‐*Prkcd*) or control lentivirus (LV‐vector) at 8 MOI (Hanbio, Shanghai, China) for 4–5 days. Cells were then subjected to immunofluorescence staining, western blot, quantitative real‐time polymerase chain reaction (qRT‐PCR), and RNA‐sequencing analyses.

### 2.7 Primary astrocyte culture

Cortical and hippocampal tissues were dissected from mouse at postnatal days 1–2 and dissociated with 0.05% trypsin for 30 min at 37°C. Tissues were centrifuged for 5 min at 500 × *g* and mechanically dissociated in DMEM/F12 (catalog #11330‐032, Gibco) medium containing 20% FBS and 1% penicillin/ streptomycin. Cells were passed through a 70 µm cell strainer, centrifuged at 500 × *g* for 3 min, and resuspended in the growth medium. Cells were plated into 175 cm^2^ flasks coated with 0.1% poly‐L‐lysine and culture for 7–10 days, and then dissociated with 0.05% trypsin and replated into six‐well plates for western blot assays.

### 2.8 Primary neuronal culture

Cortical and hippocampal tissues were dissected from mouse embryos at embryonic day 16.5 and dissociated with 0.05% trypsin and 20 U DNase for 30 min at 37°C. Tissues were then passed through a 70 µm cell strainer, centrifuged at 500 × *g* for 5 min, and mechanically dissociated in DMEM medium containing 10% FBS and 1% penicillin/ streptomycin to obtain a homogeneous single‐cell suspension. Cells were plated at a density of 1 × 10^6^ cells per well in six‐well plates and cultured for 4 h at 37°C in a 5% CO_2_ incubator. The culture medium was then replaced with a neurobasal medium (catalog #21103‐049, Thermo Fisher Scientific) containing 2% B27 (catalog #17504044, Thermo Fisher Scientific) and 1% penicillin/streptomycin. Primary neuronal culture was maintained by replacing half of the medium with fresh medium every 3 days.

### 2.9 Culture and treatment of BV‐2 microglial cells

The immortalized murine microglial BV‐2 cells were obtained from the National Platform of Experimental Cell Resources for Sci‐Tech, Cell Research Center (Beijing, China). BV‐2 cells were cultured in DMEM supplemented with 10% FBS and 1% antibiotics in a humidified incubator containing 5% CO_2_ at 37°C.

PKCδ for siRNAs and control were purchased from Sangon Biotech (Shanghai, China). The sequence of small interfering RNA (siRNA) targeting mouse PKCδ is 5′‐GCAAGAAGAACAACGGCAATT‐3′. To modulate PKCδ levels, BV‐2 cells were transfected with siRNAs targeting mouse PKCδ or lentiviral vectors expressing mouse PKCδ by using Lipofectamine 2000 (Invitrogen). After 48 h, transfected and non‐transfected cells were exposed to 10 µM Aβ1‐42, 2 µM PKCδ inhibitor rottlerin (Sigma‐Aldrich, St. Louis, MO, R5648), or both reagents for 24 h.

### 2.10 Transgenic mice and rottlerin treatment

Animals used in this study were homozygous APPswe/PS1dE9 double‐transgenic mouse lines, which harbor human APPswe (Swedish mutations K594N/M595L) and PS1 with an exon 9 deletion (PS1dE9) under the control of the mouse prion promoter, purchased from Beijing HFK Biotechnology. Animal procedures were approved by the Institutional Animal Care and Use Committee (IACUC) of Fourth Military Medical University, China, and performed in accordance with the University Policies on the Use and Care of Animals.

Six‐month‐old male APPswe/PS1dE9 mice and wild‐type (WT) littermates were divided randomly into four groups: rottlerin‐treated APPswe/PS1dE9 mice, vehicle‐treated APPswe/PS1dE9 mice, rottlerin‐treated WT, and vehicle‐treated WT mice. Male mice were used exclusively to exclude possible contributive effects from estrogen. Each rottlerin‐treated mouse received 8 mg/kg rottlerin (Sigma‐Aldrich) diluted in 2% DMSO in PBS via intraperitoneal injection once per day for 12 weeks. Concurrently, mice in the control groups were injected only with an equal volume of 2% DMSO in PBS. The treatment dose of rottlerin in this study was optimized based on previous research.^28^ During the study, the mice were free to access to food and water and housed in a pathogen‐free environment on a 12‐h light/dark cycle. Animal body weight, food and water intake, and overall general health were observed every week.

### 2.11 Morris water maze test

The Morris water maze test was performed as described previously.^13^ In brief, the test was performed in a 1.5‐m‐diameter and 0.5‐m‐high pool with a 10‐cm diameter platform placed in the southeast quadrant of the pool and water temperature was maintained at 23 ± 1°C. Trajectories of all animals were monitored and acquired by using a computerized tracking system (Water 2020; HVS Image). All animals were individually coded and randomized grouped, and all measurements were performed by an investigator blinded to group designations throughout testing.

The procedure comprised a 1‐day visible platform test, 6 days of training trials, and finally, a probe trial 24 h after the last training trial. In the visible‐platform test, mice were tested for four continuous trials, with an intertrial interval of 60 min. In the training trials, the hidden platform was located in the southeast quadrant and submerged 1.5 cm below the water surface. The mice were subjected to four trials per day for six consecutive days and given a maximum of 60 s to escape onto the hidden platform. If a mouse failed to locate the platform within 60 s, it was guided to the hidden platform by the experimenter and allowed to rest on it for 20 s, and a maximum score of 60 s was assigned. The time that an individual mouse took to reach the hidden platform was recorded as the escape latency. In the probe trial, the platform was removed, and the mice were also placed at the same starting point as in the previous trials and allowed to swim freely for 60 s. The average time that an individual mouse spent in the target quadrant that previously contained the hidden platform, was respectively recorded as a score of spatial memory.

### 2.12 Brain tissue preparation

After the behavioral tests, all animals were deeply anesthetized with sodium pentobarbital (100 mg/kg intraperitoneally) and perfused transcardially with 50 mL ice‐cold normal saline. Brains were removed and dissected through the midsagittal plane. One hemisphere was placed in 70% ethanol, followed by xylene treatment, and embedding in paraffin for immunofluorescence and laser scanning confocal microscopy. The entire hippocampus and cerebral cortex were quickly isolated from remaining hemisphere on ice, and snap‐frozen in liquid nitrogen and stored at ‐80°C to be used for the biochemical measurements. Whole brain, cerebral cortex, and hippocampus weights were measured.

### 2.13 RNA‐seq

Total RNA from primary microglial cells overexpressing PKCδ was extracted by using TRIzol (Invitrogen) according to the manufacturer's protocol and subjected to RNA‐seq analysis (BGI Genomics). Briefly, total RNA was dispensed in buffer and followed by the steps including reverse transcription, pre‐amplification, cDNA purification, and construction of cyclized ssDNA libraries. Each library was labeled with a barcode in the PCR step and was then sequenced on a BGISEQ500 sequencer with 100‐bp single‐end reads. The data were analyzed by Dr. Tom (BGI Genomics).

### 2.14 qRT‐PCR

Total RNA was extracted from BV‐2 cells, mouse brain tissue, and primary microglial cells by using TRIzol (Invitrogen) according to the manufacturer's protocol. Messenger RNA (mRNA) was reverse transcribed by using a Prime Script Double Strand cDNA Synthesis kit (Sangon Biotech, Shanghai, China), and first‐strand products were used as PCR templates. Quantitative RT‐PCR was performed by using a QuantiNava SYBR Green PCR kit and by using the iQ5 multicolor RT‐PCR detection system (Bio‐Rad Laboratories). The thermal cycling profile was as follows: 55°C for 30 min, 95°C for 15 min, and then 40 cycles of 95°C for 30 s, and 55°C for 30 s. The primer sequences used were as follows:

TNF‐α forward, 5′‐CTCCAGGCGGTGCCTATG‐3′
TNF‐α reverse, 5′‐GGGCCATAGAACTGATGAGAGG‐3′;
IL‐1β forward, 5′‐GCACACCCACCCTGCA‐3′
IL‐1β reverse, 5′‐ACCGCTTTTCCATCTTCTTCTT‐3′
IL‐6 forward, 5′‐TCCAGAAACCGCTATGAAGTTC‐3′
IL‐6 reverse, 5′‐CACCAGCATCAGTCCCAAGA‐3′;
Ccl5 forward, 5′‐TGCTCCAATCTTGCAGTCGT‐3′
Ccl5 reverse, 5′‐ GCAAGCAATGACAGGGAAGC‐3′
Cxcl3 forward, 5′‐GAAAGGAGGAAGCCCCTCAC‐3′
Cxcl3 reverse,5′‐ACACATCCAGACACCGTTGG‐3′
Ccr1 forward,5′‐GGCTTCAAAGCATGACCAGC‐3′
Ccr1 reverse,5′‐AAGCTTGCACATGGCATCAC‐3′
Tlr4 forward, 5′‐AGATCTGAGCTTCAACCCCTTG‐3′
Tlr4 reverse, 5′‐GGTGGTGTAAGCCATGCCA‐3′
Cd40 forward,5′‐GATTTGTGCCAGCCAGGAAG‐3′
Cd40 reverse,5′‐GGTGCCCTCCTTCTTAACCC‐3′
Tnfs14 forward,5′‐ATCAGGACCATGTTGGCAGG‐3′
Tnfs14 reverse,5′‐GTGGCTGGAAACCAATGCAG‐3′
β‐actin forward, 5′‐GGAGATTACTGCCCTGGCTCCTA‐3′
and β‐actin reverse, 5′‐GACTCATCGTACTCCTGCTTGCTG‐3′.

### 2.15 Western blot

Proteins were extracted from BV‐2 cells, mice brain tissues, primary microglial cells, neurons, and astrocytes in RIPA buffer containing a cocktail of protease and phosphatase inhibitors. Samples were used for western blot analyses as described previously.^13^, ^29^ In brief, samples were diluted in 4×SDS‐sample buffer, boiled, and separated by 6%–12% SDS‐PAGE and subsequently transferred to polyvinylidene fluoride membranes. Non‐specific binding sites were blocked in PBST containing 5% non‐fat dried milk for 1 h, and membranes were probed with the following primary antibodies diluted in the blocking medium overnight at 4°C: mouse anti‐PKCδ (1:500; Santa Cruz Biotechnology, Inc.), mouse anti‐IκBα (1:1000; Cell Signaling Technology), rabbit anti‐pIκBα (Ser32/36; 1:1000; Abcam), mouse anti‐NF‐κB p65 (1:1000; Cell Signaling Technology), rabbit anti‐NF‐κB p‐p65 (Ser536; 1:1000; Cell Signaling Technology), and mouse anti‐β‐actin (1:5000; Sigma‐Aldrich). Membranes were washed with Tris‐buffered saline at 0.1%, followed by incubation with horseradish peroxide‐conjugated second antibodies (1:5000; Santa Cruz Biotechnology, Inc.) for 2 h at room temperature. Signals were developed using an enhanced chemiluminescence kit (EMD Millipore), and relative band intensity was normalized relative to β‐actin using an automated image analysis system (Olympus).

### 2.16 Immunofluorescence staining

Immunofluorescence staining was performed as described previously.^13^, ^29^ Briefly, sagittal brain sections (15‐µm‐thick) were deparaffinized and rehydrated. Antigen retrieval was performed by 10 mM sodium citrate solution (pH 6.0) for 30 min at 90°C in a water bath. Non‐specific binding sites were blocked by incubation with 0.1% Triton X‐100 and 2% bovine serum albumin (BSA; Sigma‐Aldrich) for 1 h at room temperature. Sections were then incubated with mouse anti‐PKCdelta (1: 200; Santa Cruz Biotechnology, SC‐8402); rabbit anti‐GFAP (1: 1000; catalog #Z0334, Dako), rabbit anti‐MAP2 (1: 500; catalog #8707, Cell Signalling Technology), rabbit anti‐IBA1 (1: 500; catalog #019‐19741, Wako); rabbit anti‐PKCδ (1: 100; Abcam, catalog #ab182126), goat anti‐Iba1 (1:100; Wako Chemicals, catalog #011‐27991), and rabbit anti‐NF‐κB p‐p65 (pSer536; 1:100; Cell Signaling Technology, 3033S), respectively, at 4°C overnight. Sections were then stained with fluorescently conjugated secondary antibodies at room temperature for 2 h and counterstained with DAPI (1: 800; Sigma‐Aldrich) for 10 min. All sections were washed with PBS, and images were acquired using a confocal microscope (C2 Si; Nikon) or a Leica SP8 confocal microscope and subjected to quantification with ImageJ software (National Institutes of Health [NIH}, https://imagej.nih.gov/ij/). Z‐stack confocal images were acquired with a Leica SP8 (DLS) confocal microscope. The number and somatic size of Iba1^+^ cells were manually counted using ImageJ software (NIH), and lengths of Iba1^+^ cell processes were analyzed using the Filament function in the Imaris software (Bitplane, Belfast, UK; version 9.2.0).

### 2.17 Statistical analysis

All data were collected and analyzed in a double‐blind manner. All data are presented as means ± SD. Receiver‐operating characteristic (ROC) curves were used to quantify the area under the ROC curve (AUC), and the differences in the AUC were determined using DeLong statistics. The group differences in escape latency and swimming speed in the Morris water maze tests were analyzed by two‐way analysis of variance (ANOVA) with repeated measures followed by post hoc least significant difference tests for multiple comparisons. All other data were analyzed with a one‐way ANOVA followed by post hoc least significant difference or Student's *t*‐tests. Correlations between the measures of cognitive function and inflammatory biomarkers in human and transgenic mice were analyzed by Pearson's correlation coefficients. All statistical analyses were performed by using SPSS 26.0 software (SPSS), and *p* values < 0.05 were considered statistically significant.

## 3 RESULTS

### 3.1 Participant Characteristics

A total of 67 participants were enrolled and received analysis of CSF AT(N) biomarkers in this study, including 32 dementia patients with A+T+(N) (AD group) and 35 normal cognition participants with A‐T‐(N) (NC group). Demographic and clinical characteristics of the participants were summarized in Table S1. Age, sex, and education did not differ significantly between the NC group and AD group, whereas MMSE, ADL, and NPI scores, as well as levels of Aβ42, p‐Tau181, and t‐Tau in CSF (Figure S2) all presented remarkable differences between the two groups.

### 3.2 PKCδ levels are elevated in the CSF of AD patients and positively correlated with neuroinflammation

**FIGURE 1:**
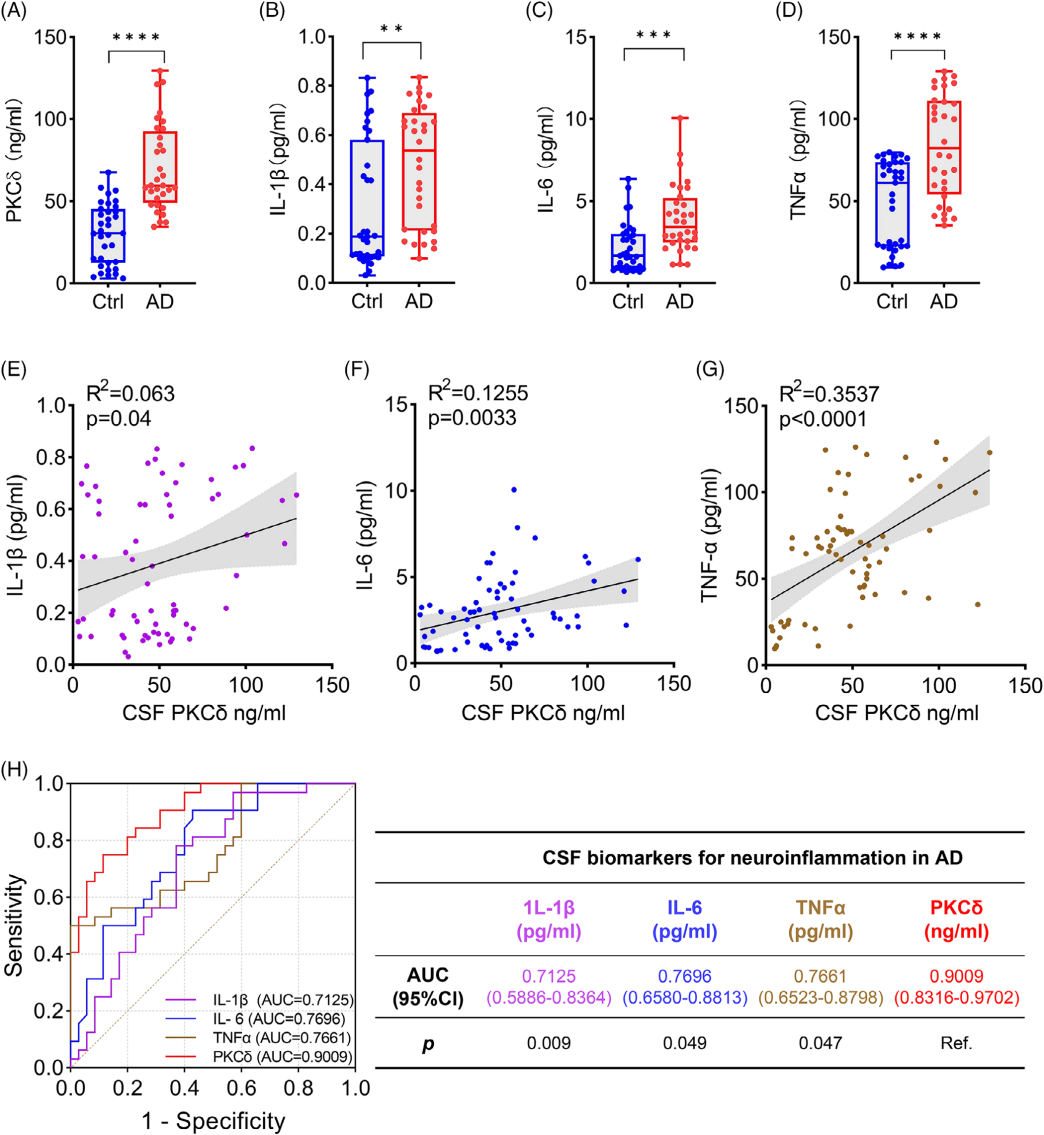
PKCδ and cytokines levels are correlatively elevated in the CSF of patients with AD. (A–D) Levels of PKCδ (A), IL‐1β (B), IL‐6 (C), and TNF‐α (D) in CSF were determined by ELISA. Data represent median (interquartile range). The midline of the box plots indicates the median and the box indicates the 25th and 75th percentiles. Unpaired *t*‐test. (E–G) Correlations between PKCδ and IL‐1β (E), IL‐6 (F), and TNF‐α (G), analyzed by linear regression with 95% CI and Pearson's correlation coefficients. (H) Receiver‐operating characteristic (ROC) curves are used to quantify the area under the ROC curve (AUC) of PKCδ (AUC = 0.9009), IL‐1β (AUC = 0.7125), IL‐6 (AUC = 0.7696), and TNF‐α (AUC = 0.7661). Differences in the AUC were determined by DeLong statistics. ** *p* < 0.01; *** *p* < 0.001; **** *p* < 0.0001. AD, Alzheimer's disease; CI, confidence interval; CSF, cerebrospinal fluid; ELISA, enzyme‐linked immunosorbent assay; IL‐1β, interleukin 1 beta; IL‐6, interleukin 6; PKCδ, protein kinase C delta; ROC, receiver‐operating characteristic; TNF‐α, tumor necrosis factor alpha.

We performed ELISA to determine protein levels of PKCδ and inflammatory cytokines that have been linked to AD in the CSF samples.^24^, ^30^, ^31^, ^32^ The results showed that levels of PKCδ, IL‐1β, IL‐6, and TNF‐α were all significantly increased in the CSF of AD patients compared with NCs (Figure 1A–D). Furthermore, PKCδ levels were positively correlated with levels of the tested cytokines in all CSF samples (Figure 1E–G). ROC analysis showed that the AUC of PKCδ (AUC = 0.9009) was significantly larger than that of TNF‐α (AUC = 0.7661), IL‐1β (AUC = 0.7125), and IL‐6 (AUC = 0.7696), indicating that PKCδ performs better than classical inflammatory proteins in distinguishing AD from non‐AD subjects (Figure 1H). Together, these results indicate that PKCδ may serve as a neuroinflammatory biomarker for AD and be involved in AD‐associated neuroinflammation.

### 3.3 PKCδ correlates with inflammatory cytokines and cognitive deficits in APPswe/PS1dE9 mice

**FIGURE 2:**
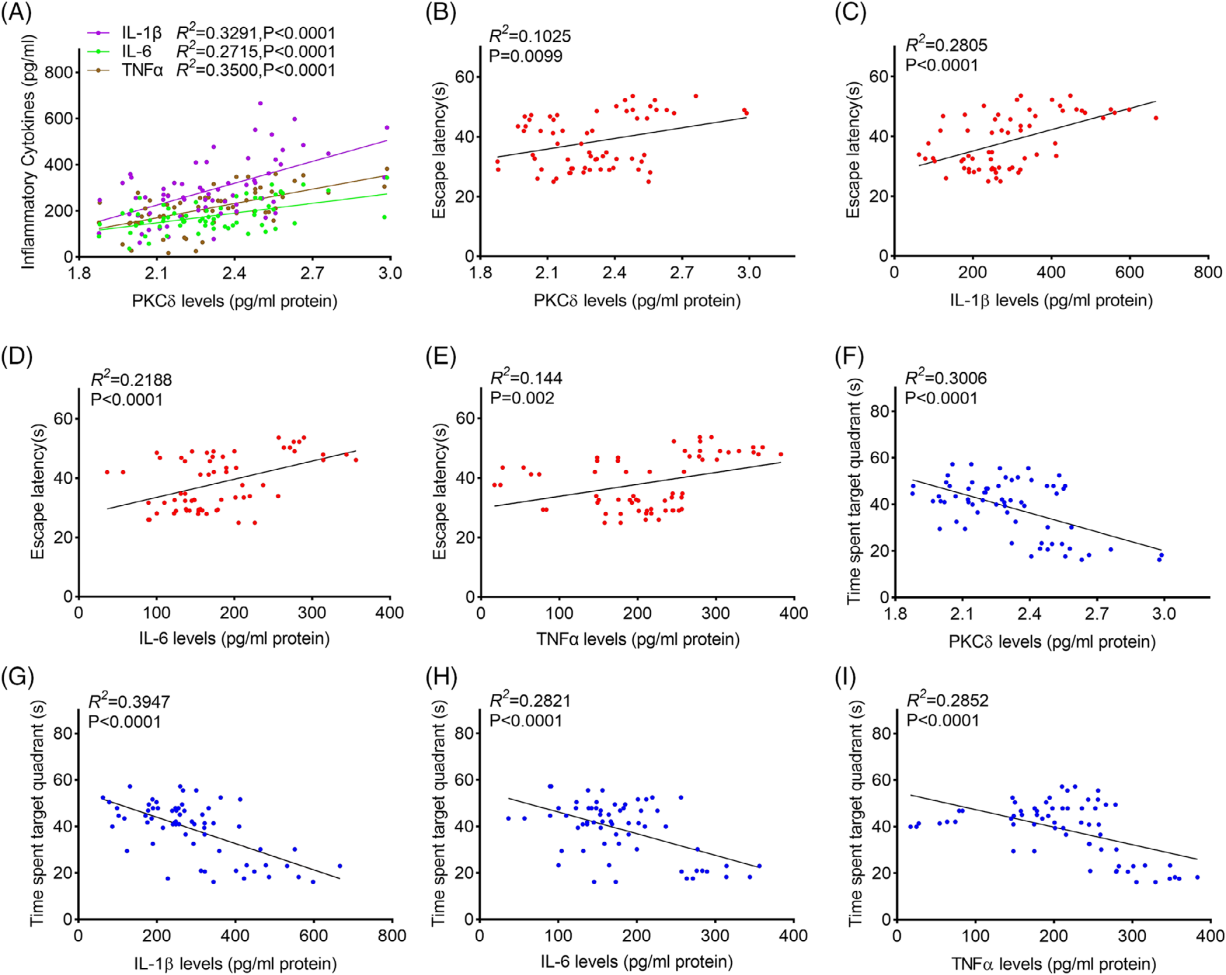
Correlations between PKCδ and inflammatory cytokines, and cognitive function in WT and APPswe/PS1dE9 mice. (A) PKCδ levels positively correlate with levels of IL‐1β, IL‐6, and TNF‐α in brain samples of APPswe/PS1dE9 mice. (B–E) PKCδ (B), IL‐1β (C), IL‐6 (D), and TNF‐α (E) levels positively correlate with escape latency on the last day of training in the Morris water maze test for spatial learning. (F–I) PKCδ (F), IL‐1β (G), IL‐6 (H), and TNF‐α (I) levels negatively correlate with time spent in target quadrant during probe test of Morris water maze test. *n* = 32 mice per group. Correlations were analyzed by linear regression and Pearson's correlation coefficients. IL‐1β, interleukin 1 beta; IL‐6, interleukin 6; PKCδ, protein kinase C delta; TNF‐α, tumor necrosis factor alpha.

Next, we investigated whether PKCδ is associated with neuroinflammation and cognitive impairment in an APPswe/PS1dE9 AD mouse model. The results from ELISA showed that PKCδ levels were correlated positively with levels of IL‐1β, IL‐6, and TNF‐α, respectively, in brain lysates from 6‐month‐old APPswe/PS1dE9 mice (Figure 2A). To evaluate cognitive function of the mouse, we performed Morris water maze test and found that PKCδ and the tested proinflammatory cytokines were positively associated with escape latencies of the mouse on the last training day (Day 6) (Figure 2B–E), and inversely correlated with time spent in the target quadrant in the probe test (Figure 2F–I) among all experimental mice. We observed similar correlations within the APPswe/PS1dE9 mouse group (Figure S3A–H). In sum, the above correlation data suggest that PKCδ may contribute to neuroinflammation and cognitive deficits in AD.

### 3.4 PKCδ is highly expressed in microglia and regulates microglia‐mediated inflammation and phagocytosis

**FIGURE 3:**
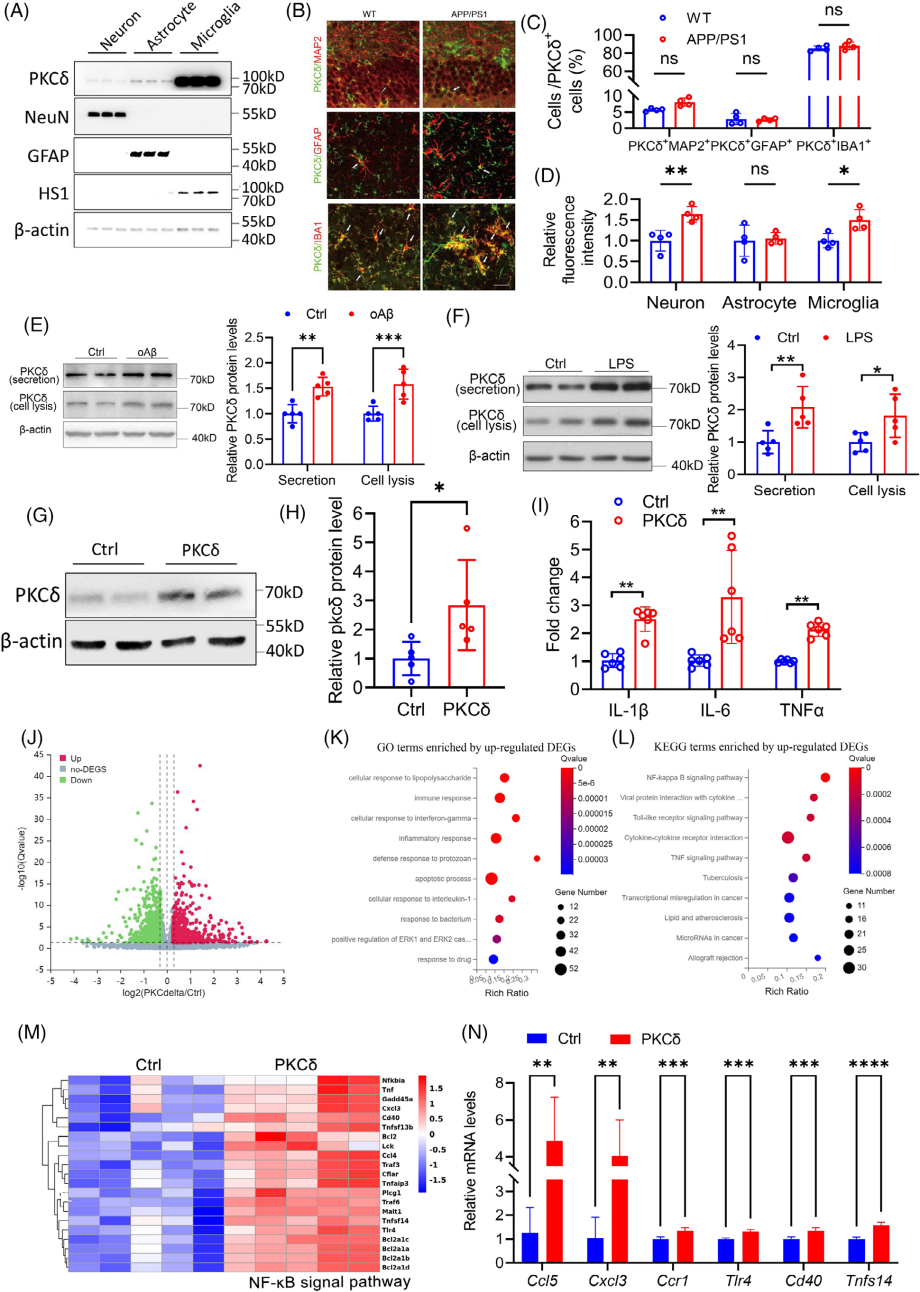
PKCδ is highly expressed in microglia and modulates microglia‐mediated neuroinflammation. (A) Western blot analysis of the expression of PKCδ and cellular markers including NeuN (for neuron), HS1 (for microglia), and GFAP (for astrocyte) in mouse primary cellular cultures. (B) Representative images of immuno‐stained PKCδ in MAP2^+^, GFAP^+^, and IBA1^+^ cells in WT and APPswe/PS1dE9 mouse brain. (C) Quantification of PKCδ^+^MAP2^+^, PKCδ^+^GFAP^+^, and PKCδ^+^IBA1^+^ cells in the brain of APP/PS1 mice relative to WT mice. *n* = 4 mice/group, two‐way ANOVA with Sidak multiple comparisons test. (D) Quantification of fluorescence intensity of PKCδ in neuronal (MAP2^+^), astrocytic (GFAP^+^), and microglial (IBA1^+^) cells in APPswe/PS1dE9 brains relative to WT controls. *n* = 4 mice/group, two‐way ANOVA with Sidak multiple comparisons test. (E) Western blot analysis of secreted and intracellular PKCδ in primary microglial culture in the presence or absence of Aβ oligomers. *n* = 5 biological replicates, unpaired *t*‐test. (F) Western blot analysis of secreted and intracellular PKCδ in primary microglial culture with or without LPS stimulation. *n* = 5 biological replicates, unpaired *t*‐test. (G–J) Primary microglia were transduced with lentiviruses for 5 days and then subjected to the analyses of western blot, qRT‐PCR, or RNA‐seq. (G, H) Western blot analysis of PKCδ expression. *n* = 5 biological replicates, unpaired *t*‐test. (I) qRT‐PCR analysis of IL‐1β, IL6, and TNFα expression. *n* = 5 biological replicates, unpaired *t*‐test. (J) Volcano plot indicating differentially expressed genes revealed by RNA‐seq; colored plots represent significantly downregulated (green) and upregulated (red) genes. Log10 Q value (y‐axis) and FC (log2FC, PKCδ vs Ctrl, x‐axis) are shown. Significance cutoffs were set to *p* < 0.05, FC > 1.2. (K) GO pathway analysis of differentially regulated genes. (L) KEGG pathway analysis of differentially regulated genes. (M) Heatmap depicting NF‐κB ‐signal‐pathway‐related genes. (N) qRT‐PCR analysis of *Ccl5*, *Cxcl3*, *Cxcr1*, *Tlr4*, *Cd40*, and *Tnfs40* expression, *n* = 5 biological replicates, unpaired *t*‐test. Scale bar = 20 µm. Data represent mean ± SD; * *p* < 0.05; ** *p* < 0.01; *** *p* < 0.001; **** *p* < 0.0001; ns, not significant. Aβ, amyloid beta; APP, amyloid precursor protein; IL‐1β, interleukin 1 beta; KEGG, Kyoto Encyclopedia of Genes and Genome; NF‐κB: nuclear factor‐kappa B; PKCδ, protein kinase C delta; qRT‐PCR, quantitative real‐time PCR.

To identify the cell types from which CSF PKCδ is mainly derived, we evaluated the expression of PKCδ in neurons, and astrocytes and microglia, two major cell types modulating neuroinflammation in the CNS. We observed highest protein levels of PKCδ in microglia among all cell types tested in primary cultures (Figure 3A). Immunofluorescence staining revealed predominant and abundant expression of PKCδ in IBA1^+^ microglial cells in the brain of WT and APPswe/PS1dE9 mice (Figure 3B,C). We detected sparse and relatively weak signals of PKCδ staining in MAP2^+^ neurons and GFAP^+^ astrocytes in mouse brain (Figure 3B,C). In addition, microglial and neuronal expression of PKCδ was significantly increased in APPswe/PS1dE9 brain (Figure 3B,D). Consistently, analysis of an AD human brain data set (GSE157827) showed that PKCδ was highly expressed in microglia in human brains, and its expression was significantly increased in neurons and microglia in post‐mortem brain samples from AD patients compared with non‐dementia controls (Figure S4). Combined analysis of 73 data sets from scREAD database^33^ demonstrate that microglia expressed higher levels of PKCδ compared to all other cell types in AD brain samples (fold change: 1.489 ± 0.415).

Next, we investigated whether PKCδ can be secreted from microglia, and we observed marked levels of PKCδ in the conditioned medium of primary microglial cultures. Because Aβ1‐42, especially its oligomeric forms, has been well established as the primary trigger to induce microglia‐mediated neuroinflammation during AD pathogenesis, we examined the effect of oligomeric Aβ1‐42 on microglial PKCδ expression and secretion. Oligomeric Aβ1‐42 upregulated both secreted and intracellular PKCδ in primary microglial cultures (Figure 3E). Treatment of microglia with LPS, a widely used inflammatory inducer, also showed similar effects on PKCδ expression and secretion (Figure 3F). Taking these data together with the results shown in Figure 1, we speculate that PKCδ in the CSF is primarily derived from microglia.

We next determined whether PKCδ can regulate microglia‐mediated inflammation. Transduction of primary microglia with lentiviral particles carrying coding sequence of mouse PKCδ successfully upregulated PKCδ expression, and resulted in dramatic elevations in mRNA levels of IL‐1β, IL‐6, and TNF‐α (Figure 3G–I). In addition, overexpression of PKCδ significantly attenuated microglial phagocytosis of fluorescent microspheres or oligomeric Aβ (Figure S5A–C). These results indicate that PKCδ regulates multiple functions of microglia. To explore the underlying mechanism, we performed RNA‐seq analysis and identified 699 upregulated, differentially expressed genes (DEGs), with Padj < 0.05 and fold change threshold of 1.2 (Figure 3J). Gene ontology (GO) enrichment analysis of upregulated DEGs showed that numerous immune‐related pathways were activated after PKCδ upregulation, whereas metabolic pathways including lipid metabolic process and cholesterol metabolic process were downregulated (Figure 3K, Figure S5D). Kyoto Encyclopedia of Genes and Genome (KEGG) enrichment analysis revealed that multiple pathways were upregulated in primary microglia with PKCδ overexpression, where NF‐κB pathway showed the highest rich ratio. Indeed, both RNA‐seq and qRT‐PCR demonstrated that the expression of genes involved in the NF‐κB signaling pathway, such as *Nfκbia*, *Ccl5*, and *Cd40*, was significantly upregulated in PKCδ overexpressed cells (Figure 3L–N). KEGG analysis also identified that lipid‐metabolism relevant pathways were downregulated upon PKCδ overexpression (Figure S5E).

### 3.5 PKCδ regulates inflammatory response and alterations in NF‐κB signaling induced by Aβ in microglia

**FIGURE 4:**
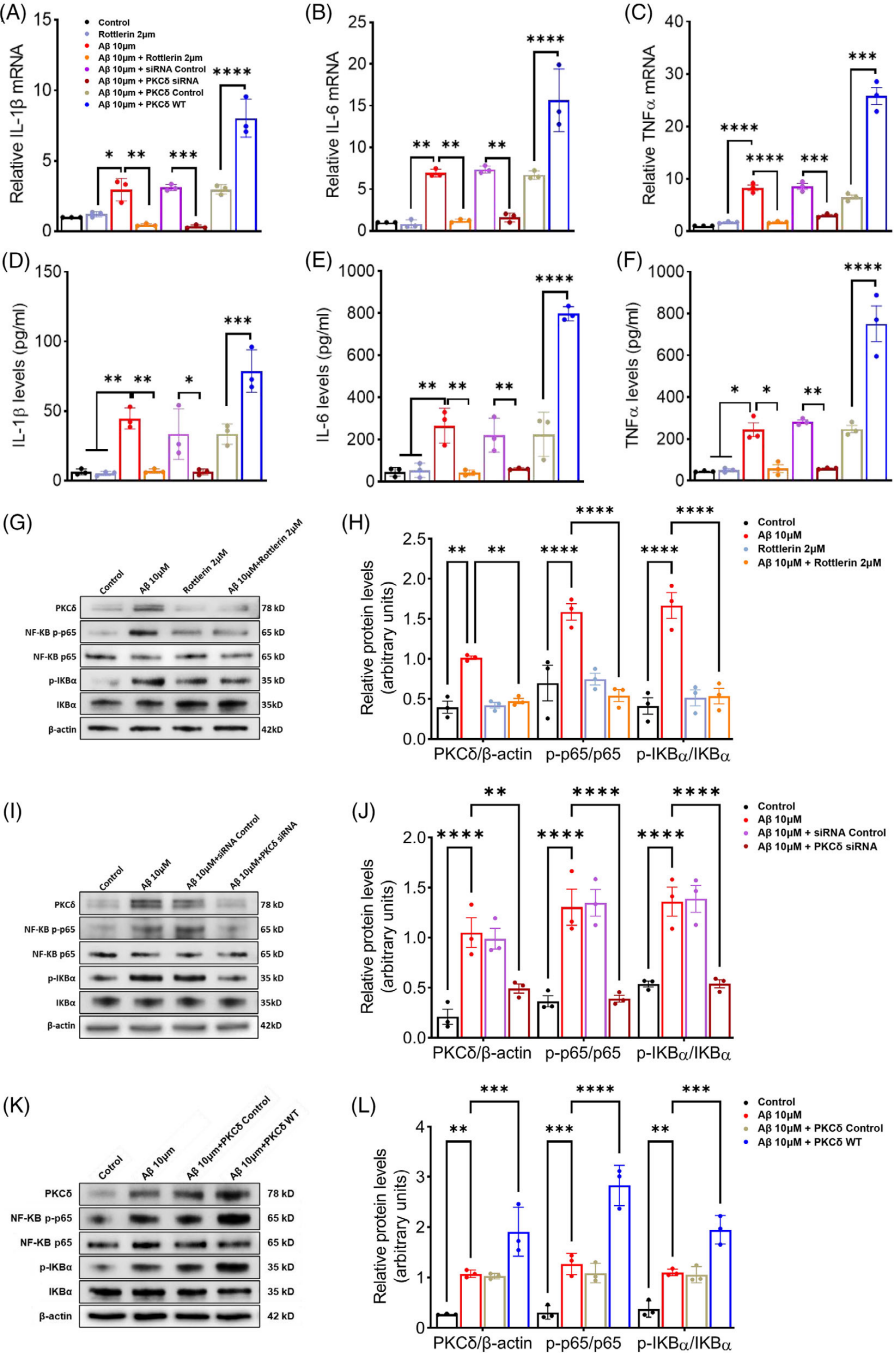
PKCδ modulates Aβ‐enhanced cytokine production and NF‐κB pathway in vitro. BV‐2 microglial cells were subjected to various treatments as indicated. (A–C) mRNA levels of IL‐1β (A), IL‐6 (B), and TNF‐α (C) were determined by qRT‐PCR. *n* = 3 biological replicates, one‐way ANOVA followed by Tukey post hoc test. (D–F) Protein levels of IL‐1β (D), IL‐6 (E), and TNF‐α (F) in the culture medium were determined by ELISA. *n* = 3 biological replicates, one‐way ANOVA followed by Tukey post hoc test. (G–L) Western blot analysis of PKCδ, p‐p65 (pSer536), p65, p‐IκBα (pSer32/36), and IκBα in cell lysates. *n* = 3 biological replicates, two‐way ANOVA followed by Bonferroni test. Data represent mean ± SD, *n* = 3 biological replicates; * *p* < 0.05; ** *p* < 0.01; *** *p* < 0.001; **** *p* < 0.0001. Aβ, amyloid beta; ELISA, enzyme‐linked immunosorbent assay; IκBα, inhibitor of kappa B alpha; IL‐1β, interleukin 1 beta; IL‐6, interleukin 6; mRNA, messenger RNA; NF‐κB, nuclear factor kappa B; PKCδ, protein kinase C delta; qRT‐PCR, quantitative real‐time PCR; TNF‐α, tumor necrosis factor alpha.

Given that PKCδ expression was increased in the microglia upon Aβ stimulation, and upregulation of PKCδ resulted in microglial proinflammatory response, we investigated whether PKCδ regulates microglial inflammation triggered by Aβ. Treatment of BV‐2 microglial cells with oligomeric Aβ1‐42 greatly increased mRNA (Figure 4A–C) and[Fig alz14047-fig-0005] protein levels of IL‐1β, IL‐6, and TNF‐α (Figure 4D–F). It is striking that these effects were fully abolished by Inhibiting PKCδ activity with rottlerin, a specific PKCδ inhibitor, or by downregulating PKCδ expression with siRNAs (Figure 4A–F). In contrast, overexpression of PKCδ further enhanced Aβ‐induced upregulation of cytokines (Figure 4A–F). These data demonstrate that PKCδ mediates Aβ‐induced microglial inflammatory response.

Because our RNA‐seq data showed that upregulation of PKCδ altered the expression of genes associated with the NF‐κB pathway, we determined changes in NF‐κB signaling in microglia with different treatments. Treatment of oligomeric Aβ1‐42 markedly increased phosphorylation of IκBα (pSer32/36) and p65 (pSer536), without affecting levels of total IκBα and p65 in BV‐2 microglial cells. These alterations were eliminated by inhibition of PKCδ (Figure 4G,H) or knockdown of PKCδ (Figure 4I,J). Conversely, PKCδ overexpression enhanced Aβ‐induced elevations in p‐IκBα (Ser32/36) and p‐p65 (Ser536) (Figure 4K,L). These results indicate that PKCδ regulates alterations in the NF‐κB pathway induced by Aβ oligomers.

### 3.6 Inhibition of PKCδ attenuates microglia‐associated neuroinflammation and improves cognitive function of APPswe/PS1dE9 mice

**FIGURE 5 alz14047-fig-0005:**
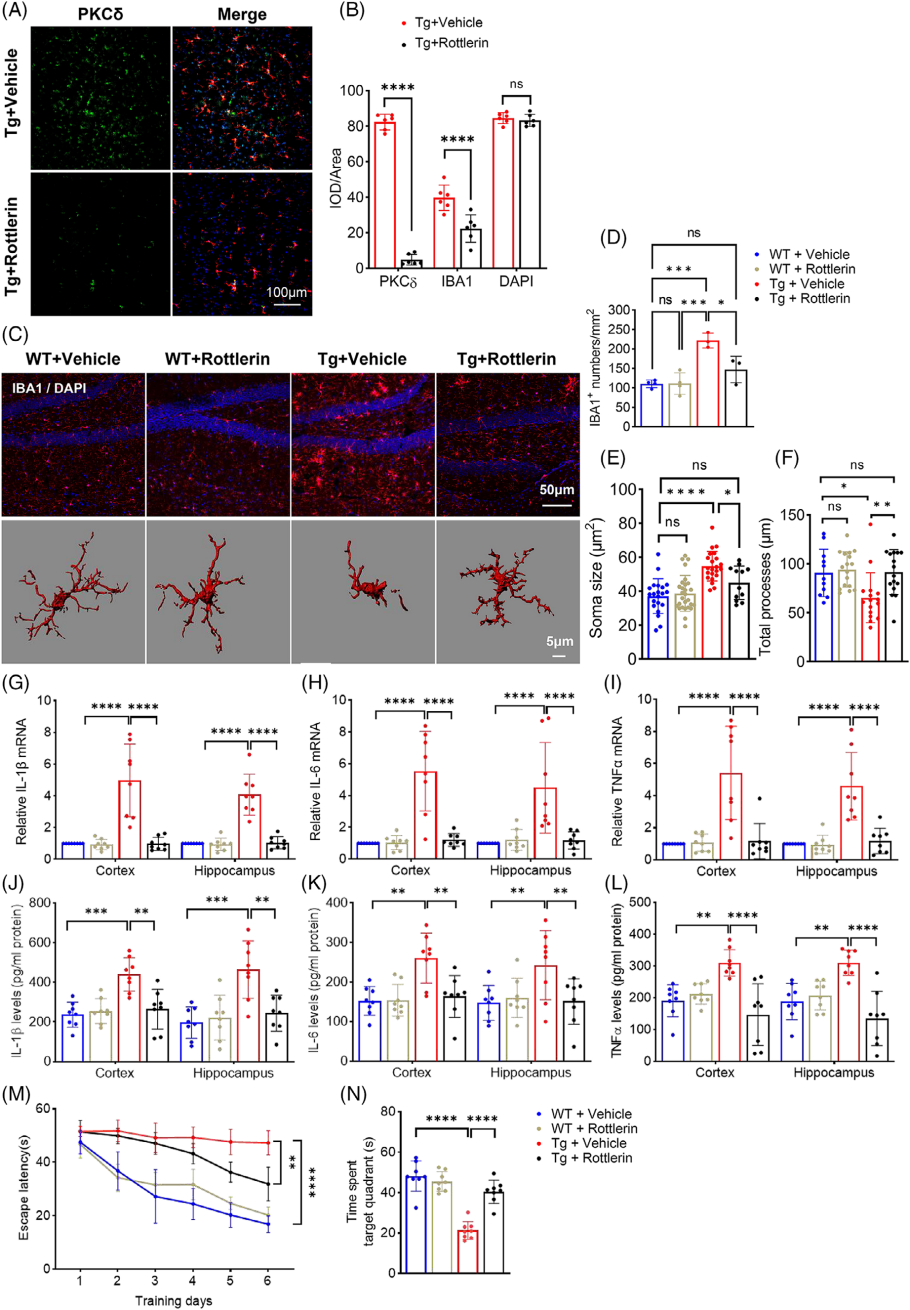
Rottlerin‐mediated PKCδ inhibition reduces microglial activation and inflammatory cytokine production and improves cognitive function of APPswe/PS1dE9 mice. (A, B) Confocal analysis of PKCδ and Iba1 in the cortex of APPswe/PS1dE9 mice. (A) representative images (Scale bar = 100 µm), (B) quantitative data, *n* = 6 mice per group, unpaired *t*‐test. (C–F) Analysis of microglial number and morphology. (C) representative images, (D–F) quantitative analysis. *n* = 3 to 4 mice per group; *n* = 11–27 microglia per group were counted, one‐way ANOVA followed by Tukey post hoc test. (G–I) mRNA levels of IL‐1β (G), IL‐6 (H), and TNF‐α (I) in cortical or hippocampal samples were determined by qRT‐PCR. *n* = 8 mice per group, two‐way ANOVA followed by Bonferroni test. (J–L) Protein levels of IL‐1β (J), IL‐6 (K), and TNF‐α (L) were determined by ELISA. *n* = 8 mice per group, two‐way ANOVA followed by Bonferroni test. (M) Escape latencies of the experimental mouse during the training phase of MWM. *n* = 8 mice per group, two‐way ANOVA with repeated measures followed by post hoc least significant difference tests for multiple comparisons. (N) Time spent in the target quadrant in the probe test of MWM. *n* = 8 mice per group, one‐way ANOVA followed by Tukey post hoc test. WT,  wild type; Tg, transgenic. Values represent mean ± SD, ** *p* < 0.01; *** *p* < 0.001; **** *p* < 0.0001; ns, not significant. ELISA, enzyme‐linked immunosorbent assay; IL‐1β, interleukin 1 beta; IL‐6, interleukin 6; PKCδ, protein kinase C delta; qRT‐PCR, quantitative real‐time PCR; TNF‐α, tumor necrosis factor alpha.

To investigate whether PKCδ mediates AD‐associated neuroinflammation in vivo, we treated 6‐month‐old APPswe/PS1dE9 (Tg) mice with rottlerin or control vehicle for 2 months and then performed various analyses. Confocal microscopy imaging showed that rottlerin treatment markedly reduced PKCδ expression in the brain of Tg mice, when compared with controls (Figure 5A,B). Within the vehicle groups, the number and soma size of Iba1‐positive microglia increased significantly, whereas the process length of microglia decreased in Tg mice compared to WT mice, indicating that microgliosis occurred at this age in AD mice. Strikingly, treatment of rottlerin eliminated microglial alterations in Tg mice (Figure 5C–F). In addition, mRNA and protein levels of IL‐1β, IL‐6, and TNF‐α significantly increased in the cerebral cortex and hippocampus in vehicle‐treated Tg mice compared with vehicle‐treated WT mice, where rottlerin treatment markedly attenuated these changes (Figure 5G–L).

We then assessed mouse cognitive function with the Morris water maze test. Vehicle‐treated Tg mice exhibited severe spatial learning and memory impairment, as demonstrated by longer escape latencies and shorter duration in the target quadrant in the probe test, when compared with vehicle‐treated WT mice. Rottlerin treatment significantly improved spatial learning and memory function in Tg mice, as evidenced by shorter escape latencies (Figure 5 M) and longer durations in the target quadrant (Figure 5N), when compared with vehicle treatment. Together, the above data demonstrate that inhibition of PKCδ blocks microglial activation and neuroinflammation in AD mice, and improves their cognitive function.

### 3.7 PKCδ inhibition reduces IκBα/p65 phosphorylation in APPswe/PS1dE9 mice

**FIGURE 6:**
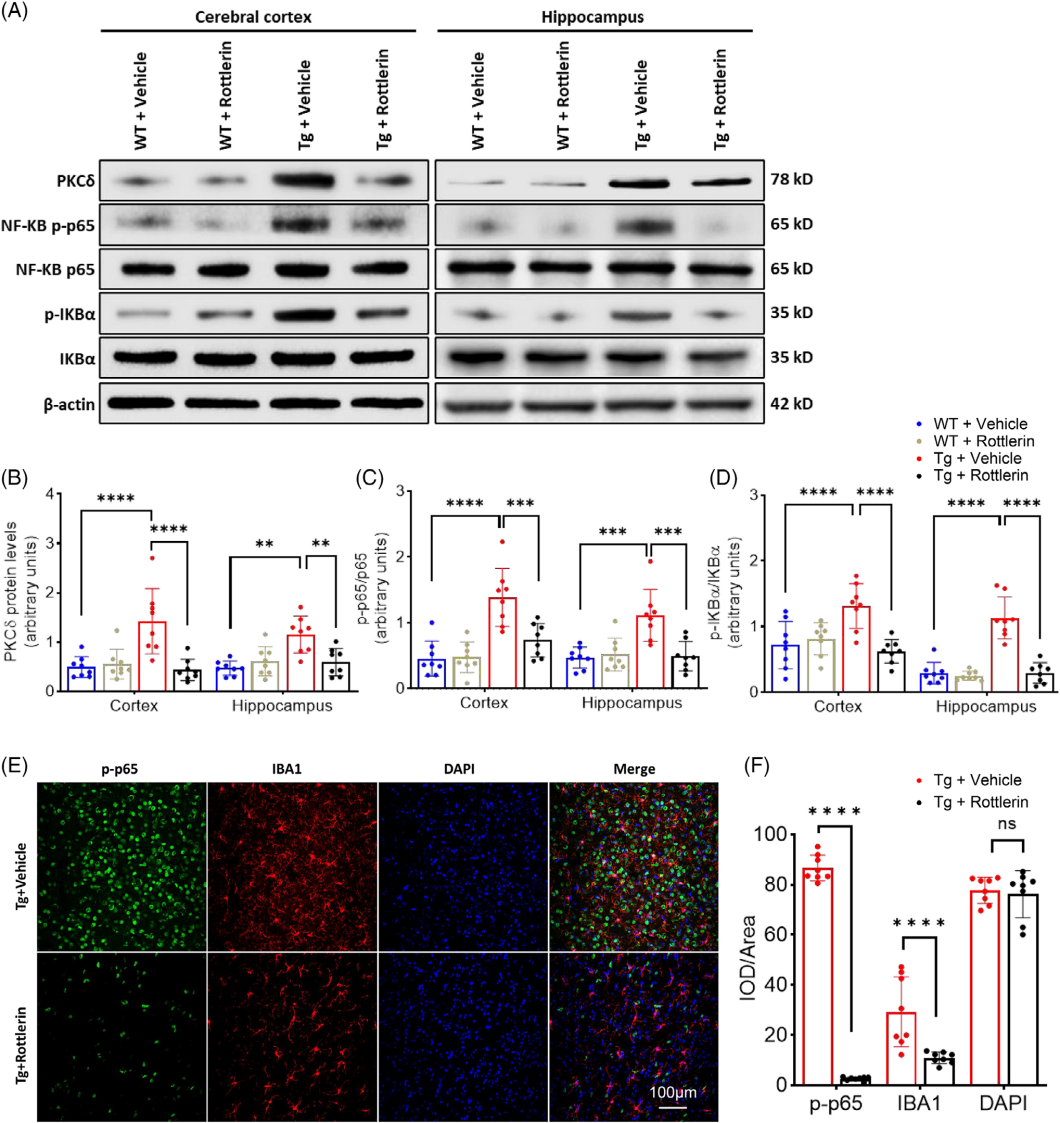
Rottlerin‐mediated PKCδ inhibition reduces NF‐κB IκBα/p65 phosphorylation in APPswe/PS1dE9 mice. (A–D) Western blot analysis of PKCδ, p‐p65 (pSer536), p65, p‐IκBα (pSer32/36), and IκBα in cortical and hippocampal samples. (A) representative images. (B–D) Densitometric quantification of protein bands. *n* = 8 mice per group, two‐way ANOVA followed by Bonferroni test. (E, F) Confocal analysis of p‐p65 in the hippocampus of APPswe/PS1dE9 mice. (E) representative images, (F) quantitative data. *n* = 8 mice per group, unpaired *t*‐test. WT,  wild type; Tg, transgenic. Values represent mean ± SD. ** *p* < 0.01; *** *p* < 0.001; **** *p* < 0.0001; ns, not significant. Scale bar = 100 µm. IκBα, inhibitor of kappa B alpha; NF‐κB, nuclear factor‐kappa B; PKCδ, protein kinase C delta.

To determine how PKCδ regulates neuroinflammation in vivo, we examined phosphoregulatory changes in major players in the NF‐κB pathway (Figure 6A). Immunoblot analyses showed that phosphorylation of p65 (Ser536) and IκBα (Ser32/36) and PKCδ protein levels were increased in the cortex and hippocampus of vehicle‐treated Tg mice, when compared to vehicle‐treated WT mice, whereas PKCδ inhibition by rottlerin reversed these alterations (Figure 6B–D). Immunofluorescence staining documented that rottlerin treatment significantly reduced levels of p‐p65 (Ser536) (Figure 6E,F), especially in the microglia of Tg mice. Collectively, our findings indicate that the NF‐κB pathway contributes to PKCδ‐mediated neuroinflammation in AD mice.

## 4 DISCUSSION

Accumulating studies have shown that neuroinflammation is commonly associated with AD brain pathology, and microglia acts as a key player in neuroinflammatory progress. Upon stimulation of toxic Aβ, the most common causal factor in AD, microglial cells are activated and release multiple pro‐inflammatory cytokines, such as IL‐1β, IL‐6, and TNF‐α, leading to neurodegenerative damages.^34^, ^35^, ^36^ In addition, previous studies demonstrated that these inflammatory cytokines were increased in the CSF of patients with A,^37^, ^38^, ^39^ indicating a potential role of neuroinflammatory factors in aiding AD diagnosis. In the present study, we confirmed the elevations of TNF‐α, IL‐1β, and IL‐6 in CSF samples from patients with AD. Moreover, we found that CSF PKCδ levels were increased in AD patients and tightly correlated with inflammatory cytokines. Importantly, CSF PKCδ shows the highest specificity and accuracy in distinguishing AD from normal subjects among PKCδ and tested cytokines. Furthermore, we demonstrated that PKCδ is highly expressed in microglia and that stimulation of Aβ oligomers upregulated the expression and secretion of PKCδ. Thus, our findings suggest that increased PKCδ in the CSF of patients with AD may be from microglia exposed to Aβ aggregates and that PKCδ may serve as a special biomarker for microglial‐mediated neuroinflammation in AD. Similar to our observations, a previous study has found secretion of full‐length PKCδ from living liver cancer cells under normal conditions and in a xenograft mouse.^40^ Mechanistically, PKCδ was secreted in an unconventional way involving SEC22B^+^ vesicles, and this process was regulated by extended‐synaptotagmin 1 (E‐Syt1), an ER‐binding protein that localizes to ER‐plasma membrane (PM) contact sites.^41^ Notably, extracellular PKCδ may have functions such as promoting growth of liver cancer cells by activation of the IGF‐1 receptor (IGF1R) and ERK1/2 signaling pathways.^40^ It would be of interest to determine whether microglial PKCδ is secreted in a similar manner and how secreted PKCδ affects neuronal systems in the future.

It has been shown that Aβ‐induced neuroinflammation contributes to cognitive dysfunction.^42^, ^43^, ^44^ In support of this notion, we found that inhibition of PKCδ by rottlerin attenuated cytokine production and improved cognitive function of AD mice. Rottlerin is a compound derived from the kamala tree *(Mallotus philippinensis)* and is considered as a relatively specific inhibitor of PKCδ. Our study and other studies have shown that rottlerin could reduce PKCδ protein levels without affecting other PKC isoforms including PKCα, β, ε, γ, and ζ under disease or stress conditions.^13^, ^45^, ^46^ Because rottlerin treatment did not alter PKCδ mRNA levels (data not shown), rottlerin may regulate PKCδ levels via post‐transcriptional mechanisms, such as modulation of PKCδ mRNA translation or regulation of PKCδ protein stability. For instance, it is possible that rottlerin can enhance intracellular degradation of PKCδ by reducing its membrane recruitment.^47^, ^48^ Several recent studies have shown that acetylpuerarin and aldose may attenuate inflammatory responses in microglia induced by Aβ25‐35 peptide or fibrillar Aβ1‐42, simultaneously accompanied with suppression of PKCδ.^49^, ^50^, ^51^ Together, these findings suggest that PKCδ is a potential therapeutic target for intervening neuroinflammation and cognitive deficits in AD. Mechanistically, we found that overexpression of PKCδ activated the NF‐κB pathway in microglia, a main downstream pathway of PKCδ signaling that has been well documented in other cell types.^12^, ^52^, ^53^ In addition, inhibition or downregulation of PKCδ reversed alterations in the NF‐κB pathway induced by Aβ in vitro and in vivo. Therefore, the NF‐κB pathway may contribute to PKCδ‐modulated neuroinflammation in the context of AD. We do not exclude the possibility that PKCδ can regulate AD‐associated neuroinflammation via other pathways, such as p38MAPK pathway that has been documented as a downstream pathway of PKCδ signaling.^11^, ^54^, ^55^

Of interest, a previous study has shown that IL‐1 could upregulate APP expression in endothelial cells and inhibition of PKC attenuated this effect.^5^ These findings suggest that PKC in other cell types may mediate the effect of cytokine on Aβ generation. It also should be noted that PKCδ has been linked to neuroinflammation in the context of other neurodegenerative conditions such as Parkinson's disease. Elevated PKCδ expression and activity, and the consequent neuroinflammatory cytokines production were observed in microglial cells exposure to MPTP and aggregated α‐synuclein.^56^, ^57^ Silencing of PKCδ expression or inhibition of PKCδ attenuated proinflammatory cytokine levels in cellular and animal Parkinson's disease models.^56^, ^57^, ^58^ Therefore, PKCδ may be a common regulator for neuroinflammation shared by neurodegenerative diseases, which requires further investigations.

A limitation of our study is that we employed only pharmacological methods to investigate the role of PKCδ in vivo. It is important to further elucidate microglial PKCδ functions using microglia‐specific PKCδ transgene or knockout mouse models in the future. Nevertheless, our data from human cohorts; from cell models with PKCδ overexpression, downregulation, or pharmacological inhibition; and from mouse models with rottlerin treatment, consistently support that PKCδ is a specific biomarker for microglia‐mediated neuroinflammation and that inhibition of PKCδ may be a viable treatment strategy for Aβ‐induced neuroinflammatory damages in AD.

## AUTHOR CONTRIBUTIONS

Wei Zhang, Yingjun Zhao, and Huaxi Xu conceived this study and designed the experiments. Ying Du, Tiantian Guo, Yunfeng Hao, and Chuan Li performed the experiments. Xia Li, Xiaoxiao Zhang, Linghui Tang, and Xia Xu performed gene silencing and overexpression in cell lines and primary microglia. Lin Li and Dan Yao performed lumbar puncture and collected cerebrospinal fluid. Huaxing Si, Jinghan Zhang, Nana Zhao, and Tong Yu performed clinical assessments of Mini‐Mental State Examination (MMSE), Activities of Daily Living (ADL), and Neuropsychiatric Inventory (NPI). Ying Du and Chuan Li analyzed the data and provided essential discussion. Yingjun Zhao and Wei Zhang wrote the manuscript.

## CONFLICT OF INTEREST STATEMENT

The authors declare that they have no competing interests to disclose. Author disclosures are available in the supporting information.

## CONSENT STATEMENT

We provided patients with detailed information about the disease and obtained the consent of the patients for lumber puncture. We have also reported to the ethical committee of Tangdu Hospital, Fourth Military Medical University, and obtained approval from the committee. All animal studies were performed according to the protocols approved by the Institutional Animal Care and Use Committee of Tangdu Hospital, Fourth Military Medical University. The data sets used and/or analyzed during the current study are available from the corresponding author.

## Supporting information

Supporting Information

Supporting Information

Supporting Information

Supporting Information

Supporting Information

Supporting Information

Supporting Information

## Data Availability

The data that support the findings of this study are available from the corresponding author, upon reasonable request.
