# Supplementary material for: PKCδ serves as a potential biomarker and therapeutic target for microglia‐mediated neuroinflammation in Alzheimer's disease
Source: Alzheimers Dement. 2024 Jun 28;20(8):5511–27. doi: 10.1002/alz.14047 (PMC11350009; doi:10.1002/alz.14047)
Supplement: Supplementary file 1 — Supporting Information [file ALZ-20-5511-s002.pdf]

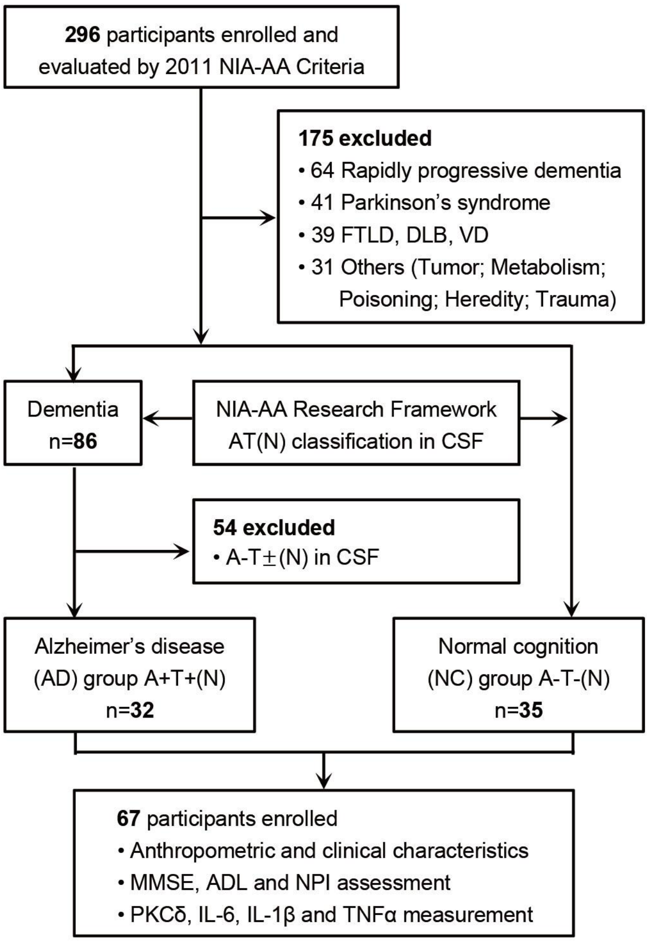

Supplementary figure 1. Flow chart of study design.

Abbreviations: NIA-AA, the National Institute of Aging and Alzheimer's Association; FTLD, frontotemporal lobar degeneration; DLB, dementia with Lewy body; VD, vascular dementia; CSF, cerebrospinal fluid; MMSE, the Mini-mental State Examination; NPI, Neuropsychiatric Inventory; ADL, Activities of Daily Living; PKCδ, protein kinase C delta; IL-1β, interleukin 1 beta; IL-6, interleukin 6; TNF-α, tumor necrosis factor alpha.
