# Supplementary material for: PKCδ serves as a potential biomarker and therapeutic target for microglia‐mediated neuroinflammation in Alzheimer's disease
Source: Alzheimers Dement. 2024 Jun 28;20(8):5511–27. doi: 10.1002/alz.14047 (PMC11350009; doi:10.1002/alz.14047)
Supplement: Supplementary file 2 — Supporting Information [file ALZ-20-5511-s004.pdf]

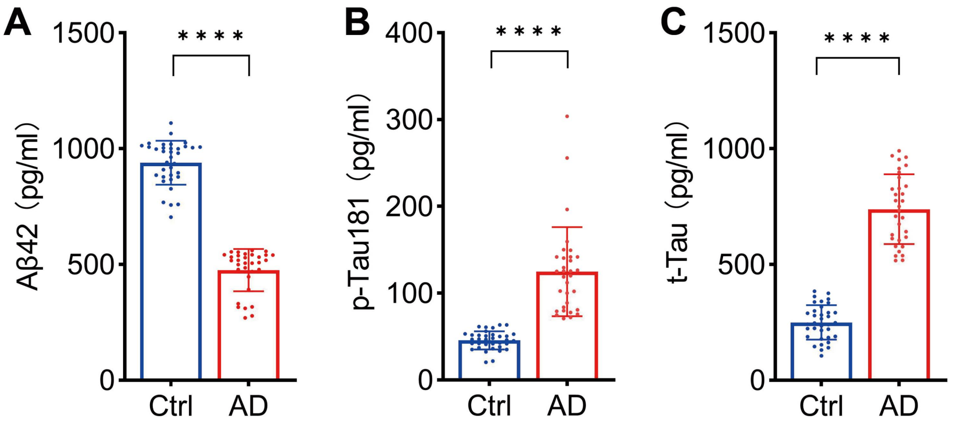

Supplementary figure 2. The level of Aβ42 is decreased and P-tau181 and T-tau levels are correlatively increased in in CSF of AD patients. Levels of Aβ42 (A), P-tau181 (B) and T-tau (C) in CSF were determined by ELISA, and student t test was used to compare the differences between AD patients and normal cognition participants, unpaired t test. Data represent mean ± SD, *p < 0.05; **p < 0.01; ***p < 0.001; ****p < 0.0001.
