# Supplementary material for: PKCδ serves as a potential biomarker and therapeutic target for microglia‐mediated neuroinflammation in Alzheimer's disease
Source: Alzheimers Dement. 2024 Jun 28;20(8):5511–27. doi: 10.1002/alz.14047 (PMC11350009; doi:10.1002/alz.14047)
Supplement: Supplementary file 3 — Supporting Information [file ALZ-20-5511-s005.pdf]

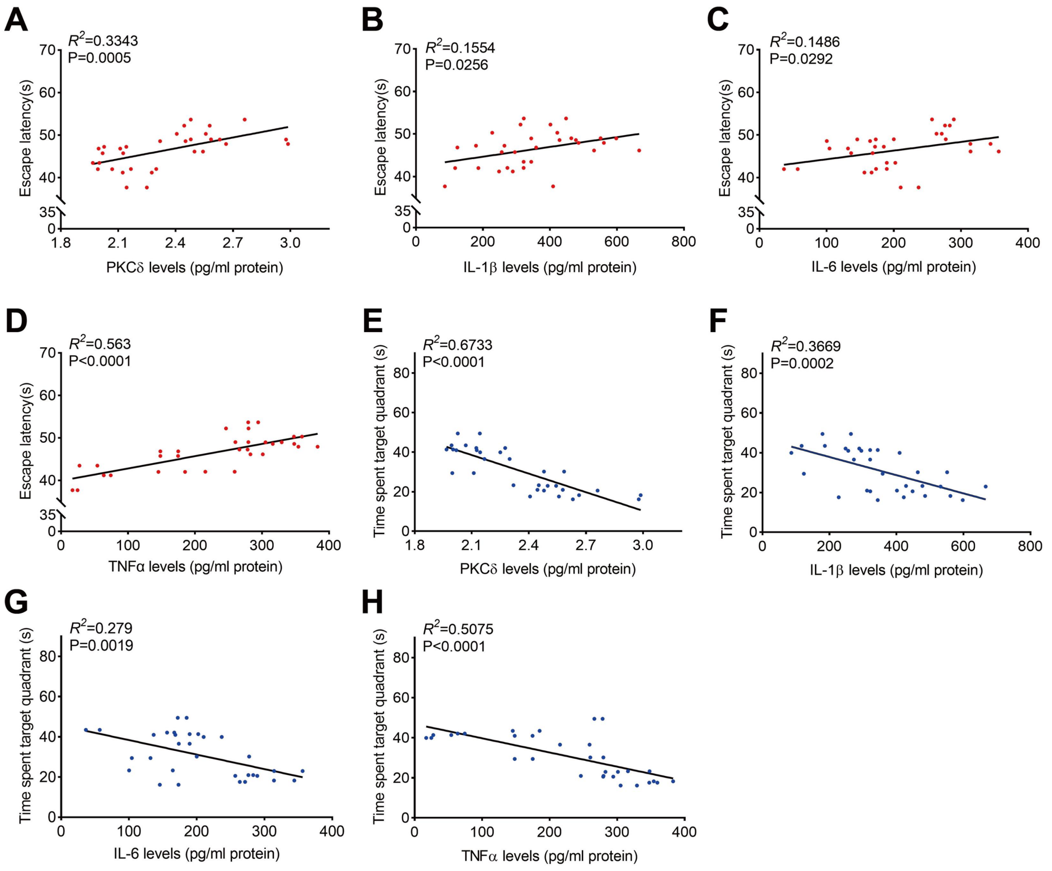

Supplementary figure 3. Correlations between PKCδ and inflammatory cytokines, and cognitive deficits in APPswe/PS1dE9 mice. (A-D) PKCδ (A), IL-1β (B), IL-6 (C) and TNF-α (D) levels in brain samples of APPswe/PS1dE9 mice positively correlate with escape latency at the last day of training in the Morris water maze test for spatial learning. (E-H) PKCδ (E), IL-1β (F), IL-6 (G) and TNF-α (H) levels negatively correlate with time spent in target quadrant during probe test of MWM test. n = 32 mice. Correlations are analyzed by linear regression and Pearson's correlation coefficients.
