# Supplementary material for: PKCδ serves as a potential biomarker and therapeutic target for microglia‐mediated neuroinflammation in Alzheimer's disease
Source: Alzheimers Dement. 2024 Jun 28;20(8):5511–27. doi: 10.1002/alz.14047 (PMC11350009; doi:10.1002/alz.14047)
Supplement: Supplementary file 4 — Supporting Information [file ALZ-20-5511-s003.pdf]

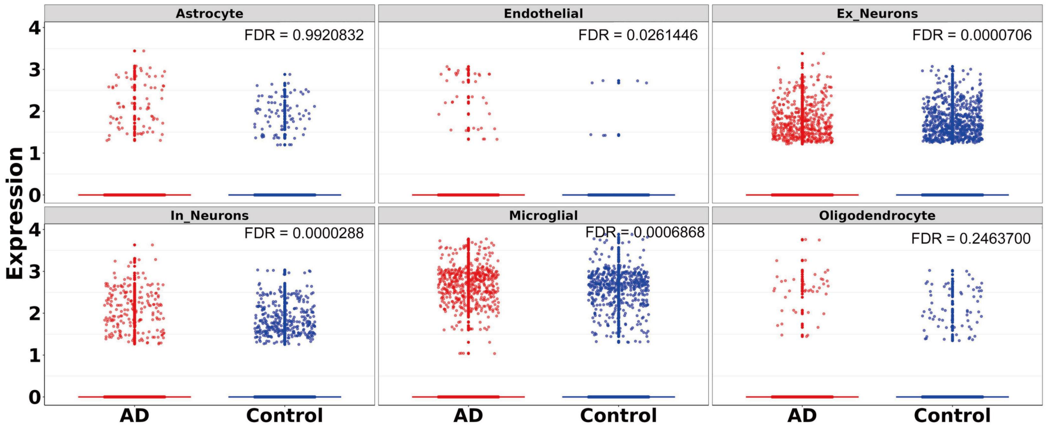

Supplementary figure 4. PKCδ is up-regulated in a variety of cells from AD patients. The expression distribution of *PRKCD* in AD and control groups (GSE157827 DataSets).
