# Supplementary material for: PKCδ serves as a potential biomarker and therapeutic target for microglia‐mediated neuroinflammation in Alzheimer's disease
Source: Alzheimers Dement. 2024 Jun 28;20(8):5511–27. doi: 10.1002/alz.14047 (PMC11350009; doi:10.1002/alz.14047)
Supplement: Supplementary file 5 — Supporting Information [file ALZ-20-5511-s001.pdf]

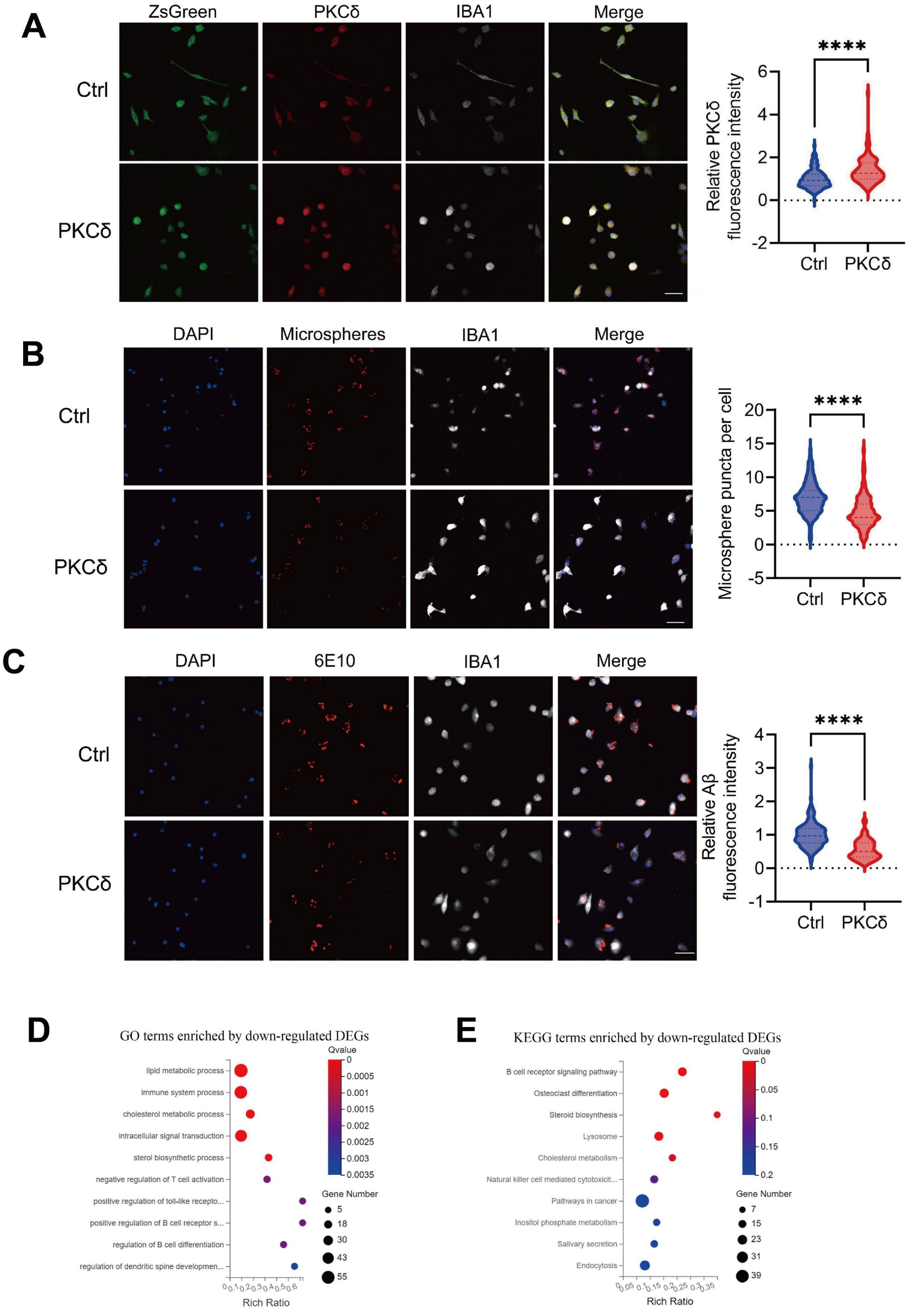


**Supplementary figure 5. Phagocytic capability of microglia is impaired and lipid-metabolism relevant pathways are downregulated upon PKCδ overexpression.** (A) Immunofluorescence analysis of PKCδ (red) expression in primary microglia (IBA1, white) transduced with LV-zsGreen-*Prkcd* or control LV-zsGreen-vector. n = 116 to 136 cells per group from 3 independent

experiments, unpaired t-test. (B) Immunofluorescence analysis of microsphere puncta (red) in primary microglia (IBA1, white) which were treated with Fluoresbrite® Polychromatic red microspheres for 2 hours. Microglial cells were transduced with LV-zsGreen-*Prkcd* or control LV-zsGreen-vector 4 days before the phagocytosis assay. n = 124 to 173 cells per group from 3 independent experiments, unpaired t-test. (C) Immunofluorescence analysis of oAβ (6E10, red) in primary microglia (IBA1, white) which were treated with 1 μM Aβ42 oligomers for 2 hours. Microglial cells were transduced with LV-zsGreen-*Prkcd* or control LV-zsGreen 4 days before the phagocytosis assay. n = 164 to 173 cells per group from 3 independent experiments were counted, unpaired t-test. (D) GO pathway analysis of differentially down-regulated genes. (E) KEGG pathway analysis of differentially down-regulated genes. Scale bar = 50 μm, ****$p < 0.0001$.
