# Supplementary material for: PKCδ serves as a potential biomarker and therapeutic target for microglia‐mediated neuroinflammation in Alzheimer's disease
Source: Alzheimers Dement. 2024 Jun 28;20(8):5511–27. doi: 10.1002/alz.14047 (PMC11350009; doi:10.1002/alz.14047)
Supplement: Supplementary file 6 — Supporting Information [file ALZ-20-5511-s006.docx]

**Supplementary Table 1.** Demographic and clinical characteristics of participants

|  | NC group (n=35) | AD group (n=32) | Statistical result |
| --- | --- | --- | --- |
| Female sex, n (%) | 45.7% | 59.4% | p=0.191 |
| Age, mean (SD), y | 64.6±7.4 | 66.93±7.4 | p=0.207 |
| Education(y), mean (SD) | 10.14±3.6 | 9.69±3.4 | p=0.601 |
| MMSE score, median (IQR) | 29(28.66, 29.34) | 11(9.16, 13.9) | p<0.001 |
| ADL score, median (IQR) | 20(20.17, 20.58) | 61(52.89, 64.05) | p<0.001 |
| NPI score, median (IQR) | 1(0.7, 1.53) | 15(15.12, 23.62) | p<0.001 |
| CSF Aβ_1-42_ (pg/ml), median (IQR) | 980.22(906.86, 972.33) | 512.77(442.33, 508.28) | p<0.001 |
| CSF P-tau181 (pg/ml), median (IQR) | 46.8(42.12, 49.38) | 122.73(106.18, 143.19) | p<0.001 |
| CSF T-tau (pg/ml), median (IQR) | 247(224.3, 275.69) | 741.9(684.31, 792.75) | p<0.001 |
| Values are n (%), mean ± SD, or median (interquartile range). Abbreviations: NC: normal cognition; MMSE: mini-mental state examination; ADL: activities of daily living scale; NPI: neuropsychiatric inventory scale; CSF: cerebrospinal fluid; Aβ42: beta-amyloid 1-42; P-tau181: phosphorylated tau 181; T-tau: total tau. | | | |
